# Hippo pathway in intestinal diseases: focusing on ferroptosis

**DOI:** 10.3389/fcell.2023.1291686

**Published:** 2023-12-06

**Authors:** Hongwei Deng, Qiuting Jia, Xin Ming, Yuxin Sun, Yuxuan Lu, Li Liu, Jun Zhou

**Affiliations:** ^1^ Department of Anesthesiology, Southwest Medical University, Luzhou, China; ^2^ Anesthesiology and Critical Care Medicine Key Laboratory of Luzhou, Luzhou, China; ^3^ School of Clinical Medicine, Southwest Medical University, Luzhou, China; ^4^ School of Basic Medicine, Southwest Medical University, Luzhou, China; ^5^ Department of Anesthesiology, The Affiliated Hospital, Southwest Medical University, Luzhou, Sichuan, China

**Keywords:** Hippo pathway, ferroptosis, intestinal diseases, mechanisms, crosstalk

## Abstract

The incidence of intestinal diseases, such as inflammatory bowel disease, gastric cancer, and colorectal cancer, has steadily increased over the past decades. The Hippo pathway is involved in cell proliferation, tissue and organ damage, energy metabolism, tumor formation, and other physiologic processes. Ferroptosis is a form of programmed cell death characterized by the accumulation of iron and lipid peroxides. The Hippo pathway and ferroptosis are associated with various intestinal diseases; however, the crosstalk between them is unclear. This review elaborates on the current research on the Hippo pathway and ferroptosis in the context of intestinal diseases. We summarized the connection between the Hippo pathway and ferroptosis to elucidate the underlying mechanism by which these pathways influence intestinal diseases. We speculate that a mutual regulatory mechanism exists between the Hippo pathway and ferroptosis and these two pathways interact in several ways to regulate intestinal diseases.

## 1 Introduction

**FIGURE 1:**
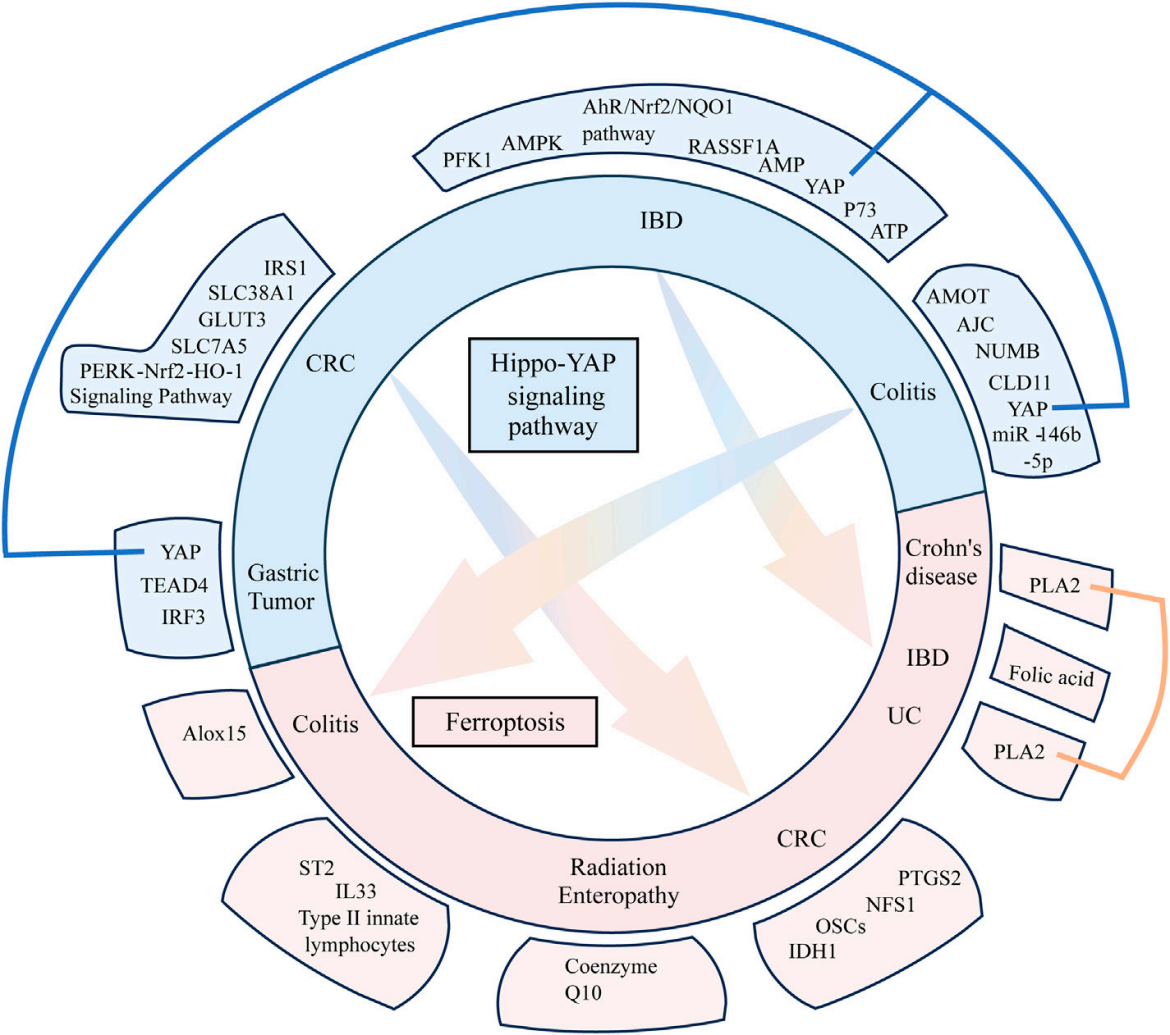
This figure visualizes the content of the disease–compound/target–mechanism table in the form of images. Three diseases, namely, colitis, colorectal cancer, and inflammatory bowel disease overlap between the two mechanisms (Hippo pathway and ferroptosis) and two overlapping targets (YAP and PLA2). The Hippo pathway and ferroptosis influence each other to some extent in terms of diseases and targets.

Intestinal diseases are becoming increasingly prevalent in society, and a large proportion of the global population is suffering from gut problems. The Hippo pathway may influence various intestinal diseases through its regulatory effect on metabolism, immunity, stem cells, and tumors. Ferroptosis is a form of programmed cell death, which contributes to the pathogenesis of several intestinal diseases. However, most of the current studies have highlighted the individual role of the Hippo pathway and ferroptosis in intestinal diseases and have not focused on the underlying relationship between these two pathways. This review summarizes the role of these two signaling pathways in intestinal diseases and explores the interconnections between them (see Figure 1). GPX4 can serve as an essential bridge between the Hippo pathway and ferroptosis, and these two pathways have opposing regulatory effects on epithelial-to-mesenchymal transition (EMT). P53 is an upstream signaling molecule that regulates these two signaling pathways; however, the subtypes that regulate them are different. In addition, gut microbes may be a potential connection between the two signaling pathways.

## 2 Hippo pathway

**FIGURE 2:**
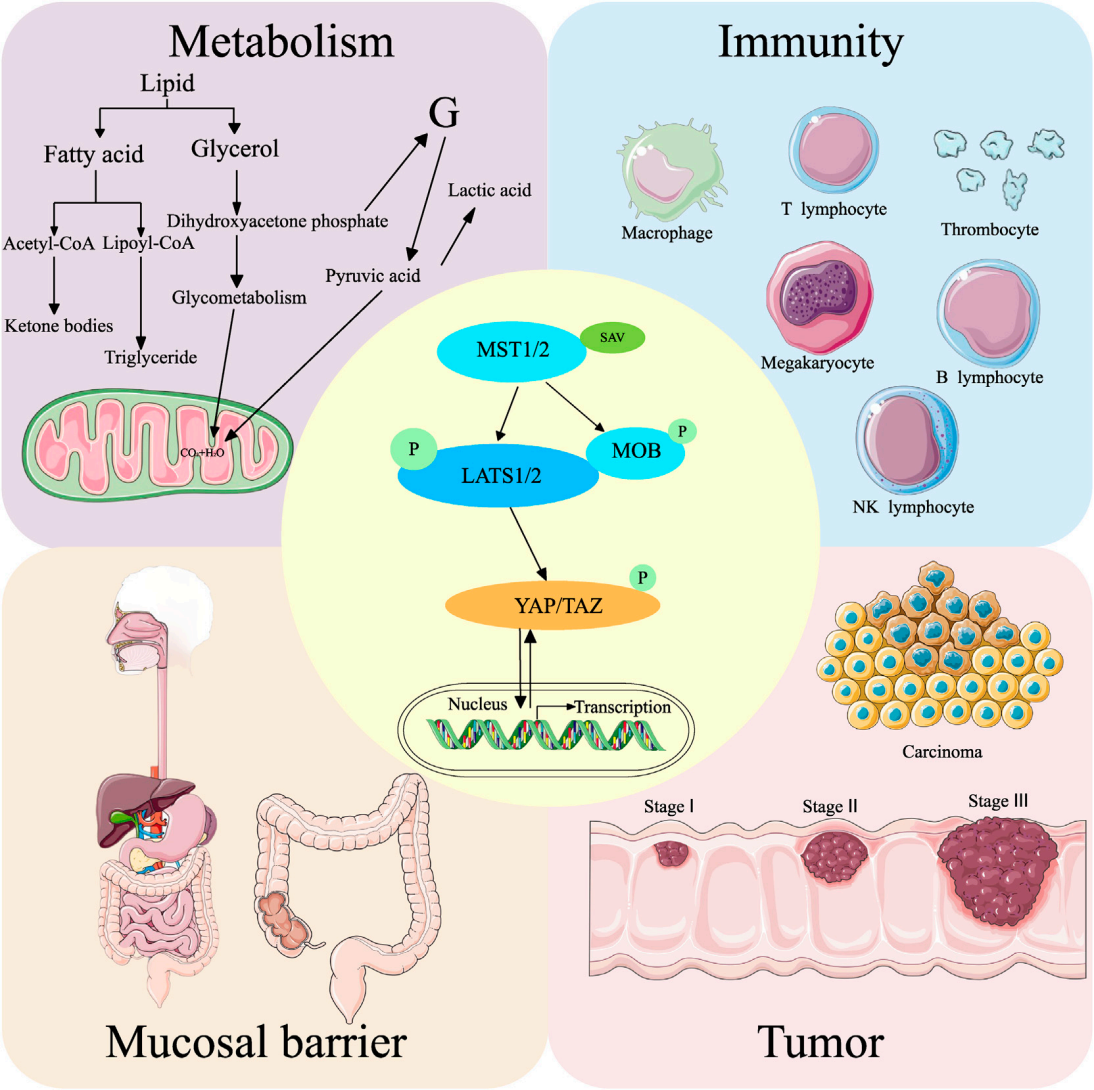
Hippo pathway and its general regulatory mechanism. MST1/2 binds to SAV to form a complex that activates LATS1/2. The activated LATS1/2 binds to MOB to activate YAP/TAZ, which regulates intracellular gene expression. The Hippo pathway regulates intestinal diseases by influencing metabolism, immunity, mucosal barrier, and tumor formation. Simultaneously, all these processes can also activate or deactivate the Hippo pathway.

The Hippo pathway was first discovered in *Drosophila melanogaster*. It comprises kinase cascade reactions, which are highly conserved from *Drosophila* to mammals. The main functions of this pathway include regulating cell proliferation, cell apoptosis, controlling organ size, affecting the development and regeneration of organisms, and regulating homeostasis (Yu et al., 2015). The core components of the mammalian Hippo pathway include the upstream large tumor suppressor (LATS) kinases (LATS1 and LATS2), mammalian Ste20-like kinases 1/2 (MST1/2), the scaffolding protein Salvador (SAV/WW45) corresponding to LATS and MST, scaffolding protein MOB domain kinase activator 1A/B (MOB1A/B), and two downstream transcriptional coactivators Yes-associated protein (YAP) and transcriptional coactivator of PDZ-binding motif (TAZ). The primary activation pathway is initiated when MST1/2 cofactor forms a complex with SAV and activates LATS1/LATS2 by phosphorylation (Hao et al., 2008). The activated LATS1/LATS2 bind to the cofactor MOB1 and phosphorylate the downstream transcriptional coactivators YAP and TAZ (see Figure 2). The subcellular location of YAP can shift between the nucleus and cytoplasm, and the phosphorylation of YAP regulates its location and expression level. The Hippo pathway influences various regulatory pathways and downstream signaling molecules. The regulatory pathways are the signaling pathways that directly phosphorylate YAP through several kinases independent of Hippo, environmental factors, immunity, cell proliferation and regeneration, cancer, and metabolism. The YAP phosphorylation, mediated by LATS, is the primary mechanism to regulate the effect of YAP protein. However, a signaling pathway that does not affect YAP through LATS has also been found. The effect of the YAP signaling pathway on intestinal diseases is partially mediated through the direct interaction of XXX with YAP. The downstream pathways of the Hippo pathway include substance metabolism, stem cell renewal and regeneration, cancer, immunity, and other metabolic processes.

### 2.1 Hippo pathway influences intestinal diseases by affecting metabolism

The Hippo pathway can influence intestinal diseases by affecting glucose, lipid, and amino acid metabolism (Table 1). This pathway modulates glucose metabolism by regulating the expression of glucose receptors, critical enzymes involved in glucose metabolism, and insulin signaling.

**TABLE 1:** Hippo pathway influences intestinal diseases by affecting metabolism and the associated mechanism.

Disease	Compound/target	Model	Effect	Mechanism	References
CRC	GLUT3	Cell	Under glucose-limited conditions, GLUT3 accelerates CRC cell growth by accelerating glucose import and promoting nucleotide synthesis	Direct transcription of the YAP–TEAD complex enhances GLUT3 expression	Wang et al. (2015), Kuo et al. (2019), Dai et al. (2020)
IBD	PFK1, AMP, ATP, AMPK	Rabbit	Promote the repair of intestinal epithelial cells	FPK1 regulates the ratio of AMP and ATP to stimulate AMPK, and the AMPK protein phosphorylates YAP at multiple sites to promote the repair of intestinal epithelial cells	Hardie et al. (2012), Mo et al. (2015), Wang et al. (2015), Arab et al. (2021), Olivier et al. (2022)
CRC	IRS1	Mouse	Worsen the prognosis of patients with CRC	TAZ stimulates IRS1 through the Wnt signaling to increase AKT activity and promote membrane localization of GLUT4	Esposito et al. (2012), Hanyuda et al. (2016), Hwang et al. (2019)
Gastric Tumor	IRF3, YAP, TEAD4	Cell	Inhibits gastric tumor growth	IRF3 interacts with YAP and TEAD4 to form a complex in the nucleus to promote nuclear translocation and activation of YAP, thereby inhibiting gastric tumor growth	Jiao et al. (2018)
CRC	SLC7A5	Mouse	Growth of KRAS-mutant CRC cells	YAP promotes the transcription of amino acid transporter SLC7A5 and enhances its expression	Najumudeen et al. (2021)
CRC	SLC38A1	Cell	Promotes the proliferation and migration of human CRC	YAP promotes the transcription of amino acid transporter SLC38A1 and enhances its expression	Zhou et al. (2017)

CRC, colorectal cancer; GLUT3, Glucose transporter 3; IBD, inflammatory bowel disease; FPK1, Fructose 1, 6-bisphosphatase; IRS1, Insulin receptor substrate 1.

In addition, the Hippo pathway can regulate glucose transporters (GLUTs). The glucose receptor subtypes regulated by YAP are still controversial. Kuo et al. reported that GLUT3 is highly expressed in colorectal cancer (CRC), and it is negatively associated with the prognosis of patients with CRC. GLUT3 promotes CRC cell growth by accelerating glucose import and promoting nucleotide synthesis (Kuo et al., 2019). However, some authors suggested that GLUT1, rather than GLUT3, is the target of YAP; therefore, further research is needed to determine the specific target of YAP.

The key enzymes in glucose metabolism have been implicated in intestinal diseases. The Hippo pathway can regulate several key enzymes in glucose metabolism, such as hexokinase 2 and phosphofructokinase/fructose bisphosphatase 3 (Zheng et al., 2017). In addition, the key enzymes in glucose metabolism can regulate YAP activity. Fructose 1, 6-bisphosphatase (PFK1) binds to the transcription factor TEAD1 through a Hippo-independent pathway and forms a PFK1–TEAD1–YAP protein complex in glycolysis (Enzo et al., 2015). In addition, PFK1 can also regulate the AMP/ATP ratio to activate AMPK (Hardie et al., 2012), which, in turn, phosphorylates YAP at multiple sites (Mo et al., 2015). The lack of AMPK inhibits the repair of intestinal epithelial cells (Olivier et al., 2022), and AMPK has been identified as a drug target for alleviating inflammatory bowel disease (IBD) in a rabbit model (Arab et al., 2021). Therefore, the repair of intestinal epithelium by AMPK may be related to YAP activity. In addition, YAP affects glucose metabolism by modulating insulin signaling. Hwang et al. indicated that TAZ can enhance the expression of insulin receptor substrate 1 (IRS1) through the Wnt signaling to increase the activity of AKT and promote the membrane localization of GLUT4 (Hwang et al., 2019). The expression level of IRS1 is associated with the physical activity and survival rate of patients with liver cancer and CRC (Hanyuda et al., 2016). This may be attributed to the increased expression of IRS1, which, in turn, increases the nuclear localization of GLUT4, and consequently carbohydrate uptake increases in tumor cells. However, further studies are needed to investigate the relationship between different GLUT subtypes (GLUT1 and GLUT3) and YAP in the context of intestinal diseases.

Adipocyte differentiation and bile acids can regulate the Hippo pathway in intestinal diseases. The Hippo pathway regulates the proliferation and differentiation of adipocytes. In contrast, the YAP protein can inhibit the differentiation of mammalian mesenchymal stem cells into adipocytes (Dupont et al., 2011). Obesity, particularly the presence of visceral fat, is markedly associated with the pathogenesis of IBD and can influence the severity and prognosis of the disease. Mesenteric adipose tissue may participate in intestinal fibrosis occurring in Crohn’s disease through the ATX–LPA axis (Huang et al., 2022). Bile acids can regulate the Hippo pathway; however, their specific role in intestinal diseases is still unclear. Primary and secondary bile acids and their oxo-derivatives have been identified as signaling molecules acting on a family of cell membrane and nuclear receptors, collectively known as “bile acid-activated receptors.” These receptors are highly expressed throughout the gastrointestinal tract and are crucial in mediating bidirectional communication between the gut microbiota and the host immune system. The expression and function of these receptors are influenced by the structure of the gut microbiota and negatively regulated by gut inflammation (Sinha et al., 2020). Simultaneously, the Hippo pathway can also affect gut microbes and intestinal diseases.

YAP promotes glycolysis and increases the transcription of amino acid transporters (AATs), thereby facilitating the uptake of amino acids in intestinal diseases. Transcription of the high-affinity AATs, such as SLC1A5, SLC7A5, and SLC38A1, is regulated by the YAP protein (Hansen et al., 2015; Park et al., 2016; Edwards et al., 2017). AATs control the movement of amino acids into and out of cells or organelles, and their dysregulation alters intracellular amino acid levels, which plays an important role in the pathogenesis of cancer, obesity, and diabetes. SLC7A5/LAT1 and SLC1A5/ASCT2 may be involved in various human malignancies (Kandasamy et al., 2018). SLC7A5 is required for the growth of KRAS-mutant CRC cells (Najumudeen et al., 2021), and SLC38A1 promotes the proliferation and migration of human CRC cells (Zhou et al., 2017).

In addition, YAP regulates protein synthesis by promoting the expression of MYC, which upregulates the expression of genes involved in ribosome biogenesis. MYC mRNA expression was increased in ileal mucosa biopsy tissues obtained from obese patients compared with those from normal individuals. Reducing MYC expression in the intestine promotes glucagon-like peptide-1 production and secretion. Therefore, an intestine-specific decrease in MYC expression in mice can ameliorate high-fat diet-induced obesity, insulin resistance, hepatic steatosis, and steatohepatitis (Luo et al., 2021). Overall, YAP can aggravate intestinal diseases and obesity by promoting the expression of high-affinity AATs and MYC.

### 2.2 Hippo pathway influences intestinal diseases by affecting immunity

Several components of the Hippo pathway, such as MST1/2, NDR1/2, and YAP/TAZ, play key regulatory roles in innate immunity. Simultaneously, the innate immune signaling also regulates the Hippo pathway. Therefore, the interaction between the Hippo pathway and the immune system is involved in the occurrence and development of tumors (Wang S. et al., 2020). We reviewed existing studies and found that this interaction is also associated with intestinal diseases (Table 2).

**TABLE 2:** Hippo pathway influences intestinal diseases by affecting immune response and the associated mechanism.

Disease	Compound/target	Model	Effect	Mechanism	References
CRC	PERK–Nrf2–HO-1 signaling pathway	Cell	Induction of ferroptosis in CRC cells	MST1/2 regulates the stability of antioxidant transcription factor Nrf2	Wei et al. (2021)
Inflammation-associated intestinal fibrosis in colitis	Nrf2 signaling pathway	Cell	Enhanced inflammation-associated intestinal fibrosis in chronic DSS-induced colitis	MST1/2 enhances inflammation-associated intestinal fibrosis in chronic DSS-induced colitis by regulating the stability of Nrf2	Wang et al. (2021)
IBD	AhR–Nrf2–NQO1 pathway	Mouse	Aggravate inflammatory bowel disease	MST1/2 regulates the stability of Nrf2 to promote IBD by inhibiting NLRP3 inflammasome activation through the AhR–Nrf2–NQO1 pathway	Wang et al. (2018)

CRC, colorectal cancer; IBD, inflammatory bowel disease.

Type I interferons (including IFN-α and IFN-β) are critical to antiviral host defenses. IFN regulatory factor 3 (IRF3) ameliorates dextran sulfate (DSS)-induced colitis. IRF3-deficient mice show lethal defects during the inflammatory and recovery phases of colitis. Additionally, IRF3 inhibits the nuclear translocation of *ß*-catenin translocation, thereby inhibiting CRC cell growth (Tian et al., 2020). IRF3 enhances the interaction between YAP and TEAD4 in the nucleus, promoting nuclear translocation and activation of YAP (Jiao et al., 2018). Furthermore, the Hippo pathway can act as an inflammatory switch. YAP inhibits the innate antiviral immune response by blocking the IFN-β signaling pathway. Conversely, the activation of innate antiviral immunity may cause YAP degradation, which helps prevent excessive inflammation and organ failure (Chen et al., 2023). In addition, MST1, a key component of the Hippo pathway, promotes the activation of IRF3 and the synthesis of IFN-β by promoting IRAK1 degradation (Li et al., 2015). IFN-β is essential to inducing broad, nonspecific resistance against viral infection by regulating the expression of multiple antiviral genes. MST1 can directly phosphorylate IRF3 at T75 and T253, which disrupts its dimerization (Meng F. et al., 2016). The bidirectional regulation of IRF3 by MST1 is a negative feedback mechanism to avoid excessive activation of innate antiviral immunity. MST1/2 activates IRF3 and induces the production of CXCL1 and CXCL2 to respond to *Mycobacterium tuberculosis* infection and alleviate peritoneal tuberculosis (Boro et al., 2016). Additionally, it may have a certain therapeutic effect on gastrointestinal mycobacterial infections.

Various toll-like receptor (TLR) ligands increase the phosphorylation of MOB1 (Geng et al., 2015), activating MST1/2 and subsequently the Hippo pathway. In addition, lipid A (ligand of TLR2), poly (I:C), and lipopolysaccharides can activate MAP4K2 (Zhong and Kyriakis, 2004), which can further activate MAPK and the Hippo pathway, indicating that the TLR ligands may interact with the Hippo pathway through MAPK. Multiple Hippo pathway agonists have been associated with intestinal diseases. The TLR1/2 agonist protospacer adjacent motif 3 (PAM3)CSK4 induces the differentiation of human and mouse monocytes into immunosuppressive M2 macrophages, suggesting that PAM3CSK4 may contribute to the prevention of colitis (Horuluoglu et al., 2020). Injection of poly (I:C), an agonist of TLR3 and TLR4, modulates the intestinal immune system in neonatal mice to ameliorate intestinal infections of *Cryptosporidium parvum* (Lacroix-Lamandé et al., 2014). Moreover, poly (I:C) enhances the efficacy of chemotherapy in paclitaxel-resistant colon cancer cells through the TLR3–UNC93B1–IFN-β signaling axis (Zhao et al., 2019) and can significantly prevent the occurrence of DSS-induced colitis (Zhao et al., 2017).

Reactive oxygen species (ROS) can activate MST. H_2_O_2_ stimulation induces the association of TRAF2 with MST1, thereby promoting the dimerization and activation of MST1 (Roh and Choi, 2016). MST1/2 can sense ROS and protect macrophages from oxidative stress by regulating the stability of the transcription factor Nrf2, which is a key transcriptional activator of the antioxidant response in macrophages. MST1/2 deficiency leads to enhanced ubiquitination of Nrf2 and reduced expression of antioxidant genes, resulting in increased oxidative stress, accelerated aging, and phagocyte death (Wang P. et al., 2019). Nrf2-related signaling pathways play a key role in inflammation and cancer development in many organs, including the gut. Nrf2 is associated with IBD (Winterbourn, 1995) and inhibits ferroptosis by regulating SLC7A11 and HO-1 to prevent acute lung injury caused by intestinal ischemia/reperfusion (Dong et al., 2020). Tagitinin C induces ferroptosis in CRC cells through the PERK–Nrf2–HO-1 signaling pathway (Wei et al., 2021). Therapeutic targeting of Nrf2 signaling by maggot extract attenuates inflammation-associated intestinal fibrosis in chronic DSS-induced colitis (Wang et al., 2021). Myristyl alcohol, a natural flavonoid, inhibits NOD-like receptor protein 3 (NLRP3) inflammasome activation through the AhR–Nrf2–NQO1 pathway, thereby attenuating IBD (Wang et al., 2018).

### 2.3 Hippo pathway influences intestinal diseases by affecting the mucosal barrier

The Hippo pathway participates in stem cell differentiation and further affects intestinal diseases. The mechanism is related to Wnt signaling, gut loss repairing, expansion of stem and progenitor cells, and inhibition of differentiation.

Wnt signaling plays a critical role in gut homeostasis (Han et al., 2020). The YAP/TAZ and Wnt signaling have overlapping functions in stem cell regulation and maintaining intestinal homeostasis. Pla2g2a inhibits the Wnt signaling by increasing YAP phosphorylation in colonic Paneth goblet cells, thereby negatively regulating the capacity of intestinal stem cells (ISCs) to form organoids (Schewe et al., 2016). Aberrant Wnt/β-catenin signaling has been frequently reported in different cancers, especially CRC (Cheng et al., 2019). The Wnt/β-catenin pathway is associated with the amelioration of DSS-induced intestinal mucosal barrier dysfunction (Dong et al., 2022). *ß*-Catenin is also involved in the interaction between YAP and Wnt. However, the role of YAP/TAZ and Wnt signaling in intestinal self-renewal still needs further study.

Intestinal damage repair is regulated by the Hippo pathway (Deng et al., 2022). YAP and TAZ are considered master sensors of the cellular microenvironment (Piccolo et al., 2014). They integrate cell polarity, physical cues, growth factors, and inflammation (Levy et al., 2008). Intestinal epithelial barrier dysfunction is a primary factor affecting susceptibility to inflammatory diseases (Groschwitz and Hogan, 2009). YAP has a significant role in the repair of the intestinal mucosal barrier (Zhao et al., 2012). The phosphorylation of YAP can be inhibited by destroying F-actin (Meng Z. et al., 2016). Several authors have suggested the relationship between F-actin and intestinal diseases. VIP, NF-κB, inducible nitric oxide synthase, and calcium can induce intestinal diseases by impairing F-actin degradation and affecting the integrity of the mucosal barrier (Ferrary et al., 1999; Banan et al., 2001a; Banan et al., 2001b; Banan et al., 2007). The mislocalization of tight junction proteins without F-actin disruption is found in inactive Crohn’s disease (Oshitani et al., 2005).

Rho proteins are the key regulators of cytoskeleton, cell morphology, and cell trafficking. Rho-GTPases are important in maintaining intestinal tissue homeostasis, especially for intestinal epithelial cells and T cells. Rho-GTPases may act as the regulators of colon cancer development. Activation and altered expression of Rho-GTPases are involved in events associated with cancer progression, such as the loss of intercellular adhesion, proliferation, migration, and invasion (Pradhan et al., 2021). Focal adhesion kinase (FAK) and its close relative Pyk2 are the non-receptor tyrosine kinases that mediate adhesion signals to promote cell proliferation, motility, and survival. FAK activates YAP signaling and induces its nuclear localization. This, in turn, activates mTOR signaling, which enhances the proliferation of transit-amplifying cells. Mice lacking FAK and Pyk2 develop spontaneous colitis with 100% penetrance at 4 weeks of age (Thomas et al., 2019). Animals treated with FAK and Src inhibitors are unable to repair large ulcers (Yui et al., 2018). This suggests that cell mechanics promoting epithelial cell migration, regulated by the Rho-GTPases/YAP and FAK/YAP signaling pathways, are essential to repairing damaged epithelial surface and intestinal mucosa. Integrin-associated FAK is selectively activated in BRAF V600E-mutated CRC cells in response to pharmacologic BRAF inhibition. FAK activation increases gene transcription, protein levels, and nuclear localization of *ß*-catenin. Vemurafenib BRAF inhibitors and *ß*-catenin or FAK small molecule inhibitors synergistically inhibit BRAF (Taylor and Schlaepfer, 2018). In addition, some authors have suggested multiple interactions between the Wnt/β-catenin and FAK signaling pathways in different cell types and organisms. Reciprocal regulation of the FAK–Wnt pathway may be a general phenomenon but has many unidentified roles in normal physiology or disease processes (Fonar and Frank, 2011).

Expansion of stem and progenitor cells and inhibition of differentiation are associated with enhanced YAP or TAZ activity (Johnson and Halder, 2014). The Notch and Wnt signaling pathways are critical to maintaining ISCs in an undifferentiated and proliferative state (van der Flier and Clevers, 2009). Notch signaling normally acts downstream of YAP, and YAP-mediated expansion of ISCs is at least partially associated with the activation of the Notch signaling pathway (Camargo et al., 2007). Inhibition of the Notch signaling can inhibit YAP-induced intestinal proliferation (Camargo et al., 2007) and results in rapid and complete conversion of all epithelial cells to goblet cells in the intestinal epithelium (Milano et al., 2004). Intercrypt goblet cells located on the luminal surface of the colon are associated with several intestinal diseases, and mice deficient in these cells have increased susceptibility to chemically induced colitis and spontaneous colitis with age (Nyström et al., 2021). Although goblet cells regulate interactions between the gut and microbes, they can become cancerous (Sinno and Jurdi, 2019).

### 2.4 Hippo pathway is involved in intestinal diseases by affecting tumors

YAP can not only promote tumor development but also inhibit tumor growth (Table 3). YAP is strongly expressed in several human cancers (Overholtzer et al., 2006). In addition to genomic amplification, YAP expression and nuclear localization increase in multiple types of human cancers (Steinhardt et al., 2008). However, YAP can also act as a tumor suppressor (Yuan et al., 2008). The oncogenic function of YAP is further supported by its upstream components, including Lats1, mob, and mer (St John et al., 1999; Lai et al., 2005). YAP exerts pro-apoptotic effects mainly through the co-activation of P73 (Strano et al., 2005). The P73 gene encodes several mRNA variants and protein isoforms, and the longest and functionally complete isoforms are P73a (mRNA) and TAP73a. Compared with normal colon tissue, the level of P73a mRNA in colon tumor tissue was reduced by 37%. The TAP73a protein levels in poorly differentiated cancer cells (G3) were five-fold higher than in moderately differentiated cells (Kotulak et al., 2016). The RAS-association domain family tumor suppressor protein 1A (RASSF1A) plays a role in repairing mucosal epithelial injury in a mouse IBD model by cooperating with the Hippo signaling molecules P73 and YAP (Nterma et al., 2020). The frequency and intensity of P73 expression were significantly higher in primary (67%) and metastatic (95%) tumors compared with those in standard clinical samples (19%) (Sun, 2002).

**TABLE 3:** Hippo pathway influences intestinal diseases by affecting tumors and the associated mechanism.

Disease	Compound/target	Model	Effect	Mechanism	References
Tumor	YAP, MYC	Mouse	Promote tumor growth in nude mice	YAP cooperates with the Myc oncogene to stimulate tumor growth in nude mice	Zender et al. (2006)
IBD	RASSF1A, P73, YAP	Mouse	Play a role in the repair of mucosal epithelial injury	RASSF1A plays a role in the repair of mucosal epithelial injury through cooperation with the Hippo pathway molecules P73 and YAP	Nterma et al. (2020)
Colitis	YAP, AMOT, miR-146b-5p, AJC, NUMB, CLD11	Mouse	Promote mucosal repair	Overexpression of YAP impairs nuclear retention of SRSF through interaction with AMOT, leading to upregulation of MALAT1 which regulates the intestinal mucosal barrier and restores intestinal homeostasis by sequestering miR-146b-5p and maintains the expression of AJC proteins NUMB and CLDN11	Wang et al. (2014), Li et al. (2021)

IBD, inflammatory bowel disease; RASSF1A, RAS-association domain family tumor suppressor protein 1A.

Tumorigenesis is directly associated with the dysregulation of the Hippo pathway (Zeng and Hong, 2008). The oncogenicity of YAP is influenced by cell polarity, and epithelial polarity controls the Hippo pathway in cancer development. Maintenance of apical–basal polarity is an important mechanism controlling mediators of signaling pathways involved in the regulation of cell proliferation, apoptosis, and differentiation. The apical polarity protein “Crumbs” can regulate YAP/TAZ. The Crumbs complex, including PALS1, LIN7c, and angiomotin (AMOT), interacts with TAZ/YAP and transmits cells by promoting TAZ/YAP phosphorylation. Crumbs3 regulates the expression of glycosphingolipids on the plasma membrane to promote colon cancer cell migration (Iioka et al., 2019). Downregulation of YAP/TAZ suppresses EMT and cancer metastasis (Bartucci et al., 2015). Notably, EMT-susceptible cell lines have constitutive defects in nuclear TAZ/YAP and Crumbs complex assembly (Varelas et al., 2010).

CTGF is the best-known target of the YAP–TEAD complex among the YAP/TAZ-induced genes. The PDZ-binding motif of YAP is required for CTGF transcription and oncogenic transforming activity (Jie et al., 2013). The expression of CTGF in the intestinal mucosa of ulcerative colitis patients correlates with the severity and grade of the disease, and increased expression of CTGF and pERK/ERK have been observed in DSS-induced wild-type mice (Song et al., 2019). However, single-nucleotide polymorphisms in CTGF did not affect the post-relapse rate after terminal ileal resection for Crohn’s disease (Burke et al., 2013).

Many cancers are associated with aberrant Wnt or Hippo pathways. Most CRCs have increased Wnt signaling (Krausova and Korinek, 2014), and approximately 85% of CRCs show increased nuclear YAP levels and its transcriptional activity (Steinhardt et al., 2008). A cross-regulatory mechanism between the Wnt or Hippo pathways and Wnt/β-catenin signaling affects the Hippo pathway. The Wnt/β-catenin target gene CD44 interacts with the Hippo YAP upstream regulator Nf2, ultimately activating the Hippo pathway (Wielenga et al., 1999; Morrison et al., 2001). ISCs express a specific CD44 variant that promotes Wnt signaling-induced intestinal tumorigenesis. In contrast, stem cells do not express the commonly expressed canonical CD44 isoform and do not promote tumor formation, indicating isoform-specific functions of CD44 in ISCs and tumorigenesis (Guo and Frenette, 2014). CD44 plays a role in the pathogenesis of intestinal diseases, and differential expression of secretory mucin MUC5AC enhances tumorigenesis and confers chemotherapy resistance through the CD44/β-catenin/p53/p21 signaling pathway (Pothuraju et al., 2020). ISCs express a specific CD44 variant that promotes intestinal tumorigenesis induced by the Wnt signaling activation (Guo and Frenette, 2014). ZMAT3, the p53-induced RNA-binding protein, is a splicing regulator that represses the splicing of oncogenic CD44 variants in CRC. However, CD44 expression in intestinal epithelium and cancer is independent of the p53 status (Zeilstra et al., 2013). YAP inhibits insulin-like growth factor (IGF) during the IGF pathway activation, which leads to the stabilization and nuclear translocation of *ß*-catenin (promoting heart development) (Xin et al., 2011). TRAF6 inhibits CRC metastasis by regulating selective autophagic degradation of CTNNB1/β-catenin and targets GSK3B/GSK3β-mediated phosphorylation and degradation (Wu et al., 2019). The inhibition or degradation of *ß*-catenin induced the downregulation of its target gene CD44, ultimately influencing intestinal diseases through the Hippo pathway.

A crosstalk occurs between the Sonic hedgehog and Hippo pathways. Inhibitors of the Sonic hedgehog signaling pathway exert anti-inflammatory effects on the intestinal epithelial cells (Ghorbaninejad et al., 2022). Coupling Hedgehog and Hippo pathways promotes stem cell maintenance by stimulating their proliferation (Huang and Kalderon, 2014). The AMOT family members have been identified as the novel substrates of LATS1/2 (Chan et al., 2013). The N-terminal region of the AMOT protein contains a conserved HXRXXS consensus site for LATS1/2-mediated phosphorylation. Overexpression of YAP impairs the nuclear retention of SRSF1 and itself through interaction with AMOT, leading to the upregulation of MALAT1 (Wang et al., 2014). MALAT1-knockout mice are hypersensitive to DSS-induced experimental colitis, and the absence of MALAT1 induces dysregulation of the intestinal mucosal barrier and disrupts intestinal homeostasis (Li et al., 2021).

Fas-active receptors induce RASSF1A to compete with RAF1 for binding to MST2 and disrupt the RAF1–MST2 inhibitory complex, thereby promoting MST2 phosphorylation of LATS1. The activated YAP translocates from the cytoplasm to the nucleus and forms a nuclear complex with P73, thereby inducing transcription of the pro-apoptotic puma gene (Matallanas et al., 2007). Notably, loss of PASSF1A synergizes with Apc (Min) to accelerate intestinal tumorigenesis (van der Weyden et al., 2008).

MOB1 is essential for spindle replication and mitotic checkpoint regulation (Hergovich et al., 2008). MOB1 activates LATS kinases in the Hippo pathway. IP-10, the human homolog of MOB1, is overexpressed in most CRCs (Zhang et al., 1997). Furthermore, reduced levels of MOB1A mRNA were detected in human CRC samples (Sasaki et al., 2007). Additionally, MOB1 phosphorylation at Thr12 was significantly decreased in cancer samples, demonstrating a strong correlation with decreased phosphorylation of YAP (Zhou et al., 2009). These findings suggest that MOB affects intestinal cancer by activating YAP.

### 2.5 Hippo pathway influences intestinal diseases by affecting mechanotransduction

Cells constantly respond to mechanical stress from neighboring cells and tension forces while migrating from the extracellular matrix (ECM) during organizational restructuring or organ development. Mechanical forces from different tissues are transmitted through membrane receptors, actin cytoskeleton, and nuclear envelope, thereby affecting gene expression within the cell nucleus. Extracellular mechanical signaling can influence intestinal diseases through the Hippo pathway, which is also associated with ferroptosis.

The extensively studied components include F-actin, mammalian MST1/2, and its co-localization with myosin. Disruption of the actomyosin stress fiber can lead to MST1/2 activation and subsequent induction of the Hippo pathway (Densham et al., 2009). F-actin regulates intestinal diseases and its association with the Hippo pathway (Section 2.3) and acts as a bridge between extracellular effects and the Hippo pathway to regulate intestinal diseases. Jin et al. analyzed cellular characteristics using machine learning and found that TFR1 staining, combined with nuclear and F-actin staining, can reliably detect apoptotic and ferroptotic cells (Jin et al., 2022). Therefore, F-actin is involved in both ferroptosis and the Hippo pathway in the context of intestinal diseases.

Notably, actin polymerization can positively regulate the Yuki/YAP activity. Stress fibers or cell morphology can also promote YAP activity in mammalian cells in a LATS-dependent manner (Sansores-Garcia et al., 2011). Diaphanous is a mammalian formin protein that promotes actin filament assembly and facilitates YAP nuclear translocation. In contrast, actin-severing components, such as gelsolin and cofilin, antagonize the function of Yki/YAP in cell growth (Aragona et al., 2013; Gaspar and Tapon, 2014). Amoebic trophozoites require a dynamic actin cytoskeleton to exert pathogenic effects in the intestines and systemic tissues. The amoebic trophozoite genome encodes several Rho-GTPases and three types of diaphanous-related formins (Bosch et al., 2012). Rho-GTPases are linked to the Hippo pathway. Therefore, the pathogenicity of amoebic trophozoites in the intestines is likely connected to the Hippo pathway. Microvillus loss is regulated by signaling pathways involving Cyclin-Dependent kinase 1 (CDK1) and Cyk-1, a protein similar to mammalian formins in a *Caenorhabditis elegans* model infected with enterohemorrhagic *Escherichia coli* (EHEC). Similar experiments were done using human intestinal cells infected with EHEC, and the results indicated that the CDK1–formin signaling axis plays a crucial role in EHEC-induced elimination of microvilli (Zheng and Pan, 2019). Inhibiting CDK1 overcomes oxaliplatin resistance in CRC by regulating acyl-CoA synthetase long-chain family member 4 (ACSL4)-mediated ferroptosis (Chen et al., 2015). The phosphorylation of TAZ by CDK1 during mitosis inhibits its oncogenic activity (Zhao et al., 2019). Therefore, both the Hippo pathway and ferroptosis are involved in the development of intestinal diseases through CDK1. Furthermore, circ_0044556 can promote colorectal carcinogenesis through the miR-665/diaphanous homolog 1 axis (George et al., 2012).

Although a correlation exists between actin and tissue growth and actin is associated with the Hippo pathway, an abnormally high expression of moesin (a protein located in the apical domain of epithelial cells that promotes actin assembly) does not induce tissue growth (Speck et al., 2003; Boggiano and Fehon, 2012). The ezrin, radixin, and moesin (ERM) protein family regulates the expression of breast cancer resistance protein and P-glycoprotein in lung, colon, and renal cancer cell lines (Schlegelmilch et al., 2011). Mouse and rats express ezrin but not moesin in their intestinal brush border epithelia. These findings indicate physiologic induction of the ERM proteins in the intestinal epithelial cells of hibernating animals and support the notion that hibernation involves differential expression of gene products that may promote cellular viability at low temperatures (Lange et al., 2015). Compared to adenomas (50%), moesin is predominantly expressed in the stroma (inflammatory cells and fibroblasts) of carcinomas (89.1%). In contrast, high expression of ezrin (indicated by a high H-score) is associated with specific adenocarcinoma types and negatively correlated with mitotic counts. However, the stroma of CRC can counter invasion through the expression of moesin. Notably, ezrin and moesin are independently expressed (Abdou et al., 2016). High moesin levels have been observed in the blood of cecal ligation and puncture-induced septic mice, and the administration of anti-moesin antibodies alleviates sepsis-associated mortality. These findings suggest that the HMGB1–RAGE–Moesin axis can trigger severe inflammatory responses (Chung et al., 2013).

The AMOT protein connects F-actin with the Hippo pathway. AMOT contains a conserved binding domain that can interact with F-actin or YAP. LATS-mediated phosphorylation of AMOT prevents its binding to F-actin but promotes its binding to YAP, resulting in the retention of YAP in the cytoplasm (Mana-Capelli et al., 2014). Section 2.4 detailed the effect of AMOT on intestinal tumors.

Integrins can also mediate between mechanical signals and the Hippo pathway. It connects to the F-actin cytoskeleton through focal adhesions on its cytoplasmic side comprising the ILK, FAK, and Src proteins. Cells increase their contraction of F-actin in response to extracellular forces and remodel their entire cytoskeleton through a process involving Rho-GTPases (such as Rho or Rac1), myosin activity, and ROCK19. This mechanical signaling engine comprising integrin/FAK/Rho-Rac/ROCK/F-actin core is a promising target for therapeutic interventions. Notably, α6β4 integrin can promote resistance to ferroptosis (Aylon et al., 2016). Rho-GTPases can influence intestinal diseases by affecting mucosal barrier function, and their association with the Hippo pathway has already been discussed in section 2.3. ILK, FAK, and Src-related strain-dependent signaling pathways mediate downstream locomotor responses. This finding suggests that inducing ILK through repeated deformation may contribute to mucosal injury recovery and mucosal barrier restoration in patients (Yuan et al., 2011). Knockdown of ILK severely affects the spreading, migration, and recovery ability of cells, which is directly correlated with reduced fibronectin deposition (Dupont et al., 2011). In addition, mechanical signal transduction enhances YAP/TAZ transcriptional response by releasing the components of the YAP/TAZ-associated SWI/SNF complex. BAF53A, an important subunit of the SWI/SNF chromatin remodeling complex, is considered a driving factor in various cancers. However, the role of BAF53A in CRC remains unknown. BAF53A is significantly upregulated in CRC tissues compared with paired adjacent normal tissues. Ectopic expression of BAF53A can promote proliferation, colony formation, and tumor development in CRC cells, whereas knocking down BAF53A impairs these cellular functions. Dual-specificity phosphatase 5 (DUSP5) is an endogenous phosphatase that specifically targets ERK1/2 and is expressed at low levels in CRC cells. BAF53A expression in CRC samples is negatively correlated with DUSP5 expression. Mechanistic studies have indicated that BAF53A interacts with P63, ultimately decreasing DUSP5 expression levels and promoting ERK1/2 phosphorylation. The BAF53A–DUSP5–ERK1/2 axis is a potential therapeutic target for CRC (Yang et al., 2022). Another subunit of the SWI/SNF complex, BCL11B, regulates intestinal adenoma and regeneration after γ-irradiation through the Wnt/β-catenin pathway (Brusatin et al., 2018).

PIEZO1, a mechanosensitive ion channel protein, promotes ovarian cancer and metastasis through the Hippo/YAP signaling axis (Butcher et al., 2009; Wolfenson et al., 2019). YAP signaling induces PIEZO1 to facilitate the proliferation of oral squamous cell carcinoma cells (Chang et al., 2018). PIEZO1 in intestinal epithelial cells mediates the inflammation in Crohn’s disease through the NLRP3 pathway (Codelia et al., 2014). Inhibiting PIEZO1 ameliorated intestinal inflammation and restricted group 3 innate lymphoid cell activation in an experimental model of colitis (Yu et al., 2012).

The neural TRP cation channel V subfamily member 1 (TRPV1)–CGRP axis regulates bone defect repair through the Hippo pathway (Jiang et al., 2023). The intestinal microbiome-derived metabolite capsaicin promotes GPX4 expression and inhibits ferroptosis in the intestine after ischemia/reperfusion injury by activating TRPV1, a pain receptor (Deng et al., 2021a). The inhibition of TRPV1 results in severe inflammation and defective tissue protection and repair processes in mouse models of intestinal injury and inflammation. Disruption of sensory damage leads to significant alterations in the gut microbiota. In addition, colonization of Gram-positive spore-forming bacteria has been observed in germ-free mice (Zhang et al., 2022).

## 3 Ferroptosis

**FIGURE 3:**
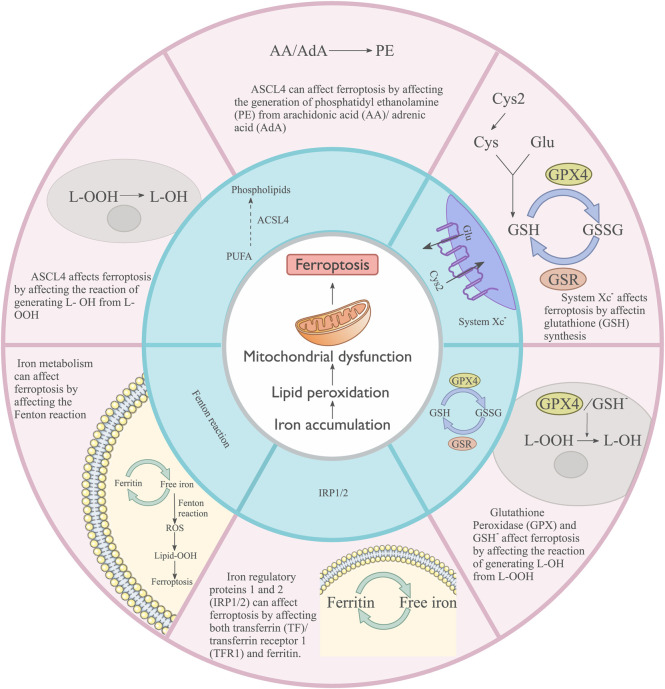
Main cellular processes influencing Ferroptosis. System XC^−^ affects ferroptosis by regulating glutathione (GSH) synthesis. Glutathione peroxidase (GPX), GSH, and ASCL4 influence ferroptosis by affecting the reaction that generates L-OH from L-OOH. ASCL4 can affect ferroptosis by affecting the generation of phosphatidyl ethanolamine (PE) from arachidonic acid (AA)/adrenic acid (ADA). Iron metabolism can affect ferroptosis by affecting the Fenton reaction. Iron regulatory proteins 1 and 2 (IRP1/2) can affect ferroptosis by affecting both transferrin (TF)/transferrin receptor 1 (TFR1) and ferritin.

Ferroptosis is associated with various cancers and diseases. Ferroptosis is a form of regulated cell death discovered in 2012, and it is distinct from previously described forms, such as necrosis, apoptosis, and autophagy (Dixon et al., 2012). The main characteristics include mitochondrial condensation and increased double-membrane density (Yagoda et al., 2007). Ferroptosis involves iron accumulation, which leads to lipid peroxidation and subsequent plasma membrane rupture (Chen et al., 2021) (Figure 3). Glutathione (GSH) homeostasis, polyunsaturated fatty acids, and cellular iron pools are involved in regulating ferroptosis.

Accumulated iron triggers ferroptosis by generating excess ROS and inducing lipid peroxidation. GSH, a key component of cellular antioxidant defense, prevents ROS accumulation (Lorenz et al., 2009). The cellular ability to use amino acids for GSH synthesis directly affects the concentration of GSH. The cells use the corresponding amino acids mainly through the system XC^−^ cystine/glutamate antiporter, which comprises heavy chain (CD98hc, SLC3A2) and light chain (xCT, SLC7A11) subunits (Ishimoto et al., 2011). GSH is a cofactor of GPX4, and its deficiency may indirectly block the GPX4 function (Ursini and Maiorino, 2020).

Plasma fatty acids are closely related to ferroptosis. Fatty acids are classified into saturated fatty acids, monounsaturated fatty acids, and polyunsaturated fatty acids (PUFAs) based on the degree of saturation of the fatty acid hydrocarbon chain. PUFAs can be oxidized to lipid peroxides (Saccà et al., 2018). ACSL4, a key regulator of lipid metabolism, is involved in regulating ferroptosis (Dherde and Krysko, 2017). ACSL4 specifically drives the esterification of arachidonic acid and adrenic acid to phosphatidylethanolamine.

Iron controls ROS generation and induces lipid peroxidation through the Fenton reaction (Winterbourn, 1995). Free cellular iron is a cofactor for lipoxygenases (LOX), a central player in ferroptosis. The processes of iron storage and export largely determine intracellular iron levels. Iron regulatory proteins 1 and 2 (IRP-1 and IRP-2) are key transcription factors in iron metabolism (Andrews and Schmidt, 2007), and both transferrin and transferrin receptor 1 (TFR1) are the potential targets of IRP1 and IRP2 (Kim et al., 1999). Ferroportin (also known as solute carrier family 40 member 1) primarily regulates iron export and is a negative regulator of ferroptosis (Ding et al., 2018). Free cellular iron can also be regulated by iron storage. Ferritin, another target of IRP1 and IRP2, binds free cellular iron and prevents ferroptosis (LaVaute et al., 2001).

### 3.1 Ferroptosis influences intestinal diseases by affecting inflammation

Effects of ferroptosis on intestinal diseases linked to inflammation are listed in Table 4. Increased expression of PTGS2 (encoding COX2) accelerates the metabolism of arachidonic acid and promotes the secretion of inflammatory mediators (Yang et al., 2014), which may enhance ferroptosis. The mRNA levels of PTGS2 were 8–9 times higher in both normal and cancerous tissues from patients with CRC compared with those in healthy individuals. Notably, carriers of the PTGS2 A-1195G variant allele have a reduced risk of developing CRC (Vogel et al., 2014). Case-control studies have shown that PTGS2 expression is associated with an increased risk of tumor recurrence and decreased CRC-specific survival in cancer patients. However, it is not associated with overall survival (Kunzmann et al., 2013). This may be due to the increased COX2 expression leading to lipid peroxidation (the main process of ferroptosis) and increased effects of substrates on the pathogenesis and prognosis of CRC. GPX4 can protect cells by inhibiting the level of cellular lipid hydroperoxides (Li et al., 2018). Some inflammatory cytokines (such as TNF, PGE2, IL-1β, IL-6, and IL-1) directly influence the level and activity of GPX4 in cancer cells (Kim et al., 2008).

**TABLE 4:** Effect of ferroptosis influences on intestinal diseases by affecting inflammation and the associated mechanism.

Disease	Compound/target	Model	Effect	Mechanism	References
Intestinal ischemia/reperfusion Injury	IL33, ST2, type II innate lymphocytes	Cell	Alleviate intestinal ischemia/reperfusion injury	Gut microbial metabolite pravastatin promotes the release of IL-13 from type II innate lymphocytes through the IL-33/ST2 signaling to alleviate intestinal ischemia/reperfusion injury	Deng et al. (2021b)
CRC	Folic acid	Clinical observation	Alleviate CRC	Folic acid alleviates CRC by inhibiting ferroptosis	Burr et al. (2017), Moazzen et al. (2018)
IBD	Folic acid	Clinical observation	Alleviate IBD	Folic acid alleviates IBD by inhibiting ferroptosis	Ratajczak et al. (2021)
CRC	PTGS2	Clinical observation	Aggravate CRC	PTGS2 encodes COX2, which accelerates arachidonic acid metabolism and aggravates ferroptosis	Kunzmann et al. (2013), Vogel et al. (2014), Yang et al. (2014)

CRC, colorectal cancer; IBD, inflammatory bowel disease.

Folic acid inhibits the ferroptosis signaling pathway (Zhang et al., 2019). Patients with IBD are at a higher risk of folic acid deficiency due to malabsorption issues and reduced intake of fresh fruits and vegetables (Ratajczak et al., 2021). A meta-analysis showed that supplemental intake of folic acid had no significant effect on CRC risk. However, total folic acid intake significantly reduced CRC risk in a cohort study (Moazzen et al., 2018). Another meta-analysis of ten studies including 4,517 patients revealed that folic acid supplementation had an overall protective effect on the development of CRC (Burr et al., 2017).

### 3.2 Ferroptosis influences intestinal diseases by affecting immunity

Macrophages constantly eliminate unnecessary cells to maintain the normal function of the body. They participate in iron homeostasis by clearing aging erythrocytes and recycling their iron. However, increased iron levels may promote ferroptosis in macrophages, thereby limiting their immune activity. Macrophages can polarize into pro-inflammatory M1 or anti-inflammatory M2 phenotype (Murray, 2017), and an imbalance in macrophage M1/M2 polarization contributes to various diseases or inflammatory conditions (Murray, 2017). Iron metabolism is also related to ferroptosis.

Neutrophils are the first immune cells recruited to sites of inflammation. They eliminate pathogens by phagocytosis, degranulation, and release of neutrophil extracellular traps (NETs), resulting in the death of infected cells. IBD has been linked to NET-induced intestinal injury and thrombosis (Li et al., 2020). In addition, NETs also maintain inflammatory signaling in ulcerative colitis (Dinallo et al., 2019) and drive EMT in cancer (Stehr et al., 2022). NETs can directly trigger the death of intestinal epithelial cells through their associated molecules (Chen et al., 2022). Activation of NADPH oxidase (NOX) and peptidylarginine deaminase 4 (most notably PAD4) is critical for NET formation (Li et al., 2010). Sulfasalazine-generated ROS promotes the non-enzymatic formation of ether-linked oxidized phospholipids, leading to the formation of NETs *in vitro* and *in vivo* (Yotsumoto et al., 2017). Accumulation of neutrophils triggers and modulates the initial inflammatory response after trauma, which may lead to subsequent tissue damage. The regulatory factors that mediate the occurrence of ferroptosis are also related to the formation of NETs; therefore, the formation of NETs may also be involved in ferroptosis. However, the relationship between ferroptosis and neutrophils in intestinal disease pathogenesis requires further studies.

The activity and function of cytotoxic T cells (CD8^+^) and helper T cells (CD4^+^) are regulated by lipid peroxidation and ferritin loss. Deleting the GPX4 gene or treatment with GPX4 inhibitors (e.g., RSL3, ML162, and ML210) induces lipid peroxidation and concomitant ferroptotic cell death in T cells *in vitro* (Matsushita et al., 2015; Drijvers et al., 2021). Conversely, the overexpression of GPX4 and Aifm2 or knockdown of Acsl4 protects CD8^+^ T cells from ferroptosis (Drijvers et al., 2021). IFN-γ secreted by CD8^+^ T cells downregulates the expression of systemic XC^−^ subunits SLC3A2 and SLC7A11, impairing the uptake of cystine by tumor cells and promoting GSH depletion secondary to ferroptosis (Wang W. et al., 2019). Ferroptotic cancer cells can be considered immunogenic and eventually activate CD8^+^ T cell-mediated antitumor immune responses (Efimova et al., 2020).

TLR4 plays a central role as a pattern recognition receptor in triggering innate immune responses. Lipid oxidation products (such as 4-hydroxynonenal and oxidized phospholipids) partly trigger inflammation *in vitro* or *in vivo* by activating TLR4 signaling (Imai et al., 2008; Wang Y. et al., 2019), suggesting that TLR4-induced inflammatory responses may be related to ferroptosis-related reaction products. TLR/MyD88 mediates innate immunity in intestinal graft-versus-host disease (Lee et al., 2017). Increased lipid peroxidation caused by GPX4 depletion limited STING1-mediated type I IFN antiviral immune responses during herpes simplex virus one infection in mice (Jia et al., 2020). However, the involvement of ferroptosis in regulating type I IFN production through STING1 in intestinal diseases remains to be studied.

### 3.3 Ferroptosis affects intestinal diseases by affecting cancer

Oncogenes of the RAS family (HRAS, NRAS, and KRAS) are the most commonly mutated genes in all human cancers (Ryan and Corcoran, 2018). RAS mutations are found in approximately half of the patients diagnosed with metastatic colorectal cancer and are associated with a poor prognosis (Patelli et al., 2021). The ferroptosis inducers (erastin and RSL3) can selectively destroy RAS-mutant tumor cells (Yang and Stockwell, 2008). However, genetic or pharmacologic inhibition of RAS or its downstream signaling molecules (BRAF, MEK, and ERK) reversed the anticancer activity of erastin and RSL3 (Yang and Stockwell, 2008). RAS-dependent and -independent mechanisms of ferroptosis were identified by analyzing 117 cancer cell lines (Yang et al., 2014). Therefore, some researchers speculate that patients with RAS mutations can be treated by inducing ferroptosis.

P53 is biallelically mutated or deleted in approximately 50% of human cancers, leading to unrestricted tumor progression (Bykov et al., 2018). It can act as a promoter to bind target genes and then activate or repress mRNA synthesis. P53-mediated transcriptional repression of SLC7A11 promotes ferroptosis in cancer cells (Jiang et al., 2015). Additionally, P53 inhibits tumor growth by affecting glucose, lipid, and amino acid metabolism and other metabolic processes (Yu et al., 2021). P53 can also inhibit NOX-mediated lipid peroxidation in human CRC cells by directly binding to dipeptidyl peptidase 4 (Xie et al., 2017). TP53 (rs1042522) and MDM2 (rs2279744) variants may represent candidate risk factors for determining susceptibility to CRC (Elshazli et al., 2020).

NFE2L2 is a master regulator of oxidative stress signaling and has dual roles in tumor progression. Insufficient NFE2L2 activity leads to early tumorigenesis, whereas overactive NFE2L2 leads to tumor progression and resistance to therapy (Rojo de la Vega et al., 2018). NFE2L2 limits oxidative damage in ferroptosis by transactivating several cytoprotective genes involved in iron metabolism, GSH metabolism, and ROS detoxification (Anandhan et al., 2020). EMT is a process by which epithelial cells lose the polarity and cell–cell adhesion properties associated with the epithelial phenotype and gradually acquire the migratory and invasive abilities associated with the mesenchymal phenotype (Yang J. et al., 2020). EMT may generate cancer stem cells, leading to metastatic spread and resistance to therapy (Yang J. et al., 2020). LYRIC (also known as Metadherin), a positive regulator of EMT, can promote ferroptosis by inhibiting the expression of GPX4 and SLC3A2 (Bi et al., 2019). Metadherin (and its variants) and the anti-metadherin antibodies are associated with CRC progression, poor prognosis, and reduced survival (Abdel Ghafar and Soliman, 2022).

### 3.4 Ferroptosis influences intestinal diseases by affecting metabolism

Multiple metabolic pathways can influence cellular susceptibility to ferroptosis (Table 5). The thiol-containing tripeptide GSH is the main antioxidant in mammalian cells and serves as the substrate of GPX4. The consumption of GSH can directly affect the activity and stability of GPX4, thereby increasing the sensitivity of cells to ferroptosis (Seibt et al., 2019). The synthesis of GSH depends on the cystine/glutamate antiporter system XC^−^ (SLC7A11), and the regulation of this system can enhance or weaken cells.

**TABLE 5:** Ferroptosis influences gut disease by affecting metabolism and its associated mechanism.

Disease	Compound/target	Model	Effect	Mechanism	References
CRC	NFS1	Cell	Phosphorylated NFS1 reduces the sensitivity of CRC to oxalate-platinum drugs	NFS1 affects CRC drug resistance by participating in iron metabolism and then affecting ferroptosis	Lin et al. (2022)
CRC	OSCs	Mouse	Promotion	OSCs affect ferroptosis by regulating the mevalonate pathway to accelerate the occurrence of CRC.	Li et al. (2022)
CRC	OSCs	Human cell sequencing analysis	Promotion	OSCs promote CRC cell proliferation by accumulating calcitriol and activating the CYP24A1-mediated MAPK signaling	He et al. (2021)
Radiation enteropathy	Coenzyme Q10	Rats	Reduce inflammation and fibrosis associated with radiation enteropathy	Coenzyme Q10 attenuates radiation-induced enteropathy by inhibiting the NF-κB/TGF-β/MMP-9 pathway	Mohamed and Said (2021)
CRC	IDH1	Cell	Inhibition	SIRT2-dependent IDH1 deacetylation suppresses CRC and liver metastasis	Wang et al. (2020b)
Crohn’s disease	PLA2	Cell	Inhibition	PLA2 can negatively regulate ferroptosis	Minami et al. (1994), Peuravuori et al. (2014)
Ulcerative Colitis	PLA2	Cell	Inhibition	PLA2 can negatively regulate ferroptosis	Minami et al. (1994), Peuravuori et al. (2014)
Colitis	Alox15	Mouse	Inhibition	Alox15 gene deficiency alleviates inflammation by suppressing ferroptosis	Kroschwald et al. (2018)

Colorectal cancer, CRC; squalene epoxidases, OSCs; isocitrate dehydrogenase, IDH; phospholipaseA2, PLA2.

Glutaredoxins are a class of GSH-dependent thiol-disulfide oxidoreductases involved in coordinating iron-sulfur clusters (Berndt and Lillig, 2017). The iron-sulfur cluster biosynthesis enzyme NFS1 cysteine desulfurase (Alvarez et al., 2017) or the mitochondrial iron frataxin (Du et al., 2020) have been associated with ferroptosis. Phosphorylated NFS1 reduces the sensitivity of CRC cells to oxalate-platinum drugs (Lin et al., 2022). Notably, the localization of frataxin is altered in the human colon adenocarcinoma cell line Caco-2 (Acquaviva et al., 2005).

LOX, specifically 12/15-LOX (ALOX15), plays a central role in lipid peroxidation and ferroptosis (Yang et al., 2016; Kagan et al., 2017). Female mice carrying a defective Alox15 gene are protected from experimental colitis through sustained maintenance of intestinal epithelial barrier function (Kroschwald et al., 2018). In addition, P53 indirectly promotes ALOX15 expression to regulate ferroptosis through its transcriptional target spermidine/spermine N1-acetyltransferase 1 (Ou et al., 2016). Moreover, the knockout of Alox15 could not rescue GPX4-deficiency-induced ferroptosis in fibroblasts (Friedmann Angeli et al., 2014).

The mevalonate pathway includes the production of isopentenyl pyrophosphate, squalene, coenzyme Q10, and cholesterol, which affect ferroptosis. Isopentenyl pyrophosphate is a precursor of squalene and coenzyme Q10 (CoQ10) and a limiting substrate for enzymatic prenylation of Sec-tRNA (Moosmann and Behl, 2004; Friedmann Angeli and Conrad, 2018). Squalene epoxidases (OSCs) drive cancer cell proliferation and promote intestinal dysbiosis to accelerate colorectal carcinogenesis (Li et al., 2022). Cholesterol accumulation decreases the concentration of OSCs, ultimately accelerating the progression and metastasis of CRC (Jun et al., 2021). The accumulation of cholesterol is related to ferroptosis and cancer (Liu et al., 2021). Therefore, OSCs may regulate ferroptosis by modulating cholesterol accumulation, thereby affecting intestinal cancer. OSCs promote CRC cell proliferation by accumulating calcitriol and activating CYP24A1-mediated MAPK signaling (He et al., 2021). MAPK can also activate the Hippo pathway to modulate intestinal diseases. FIN56, an agonist of squalene synthase SQS, acts as a ferroptosis inducer, possibly due to the depletion of CoQ10 (Shimada et al., 2016). CoQ10 attenuates inflammation and fibrosis associated with radiation enteropathy through inhibition of the NF-κB/TGF-β/MMP-9 pathway (Mohamed and Said, 2021).

Conditions that regulate glutamine cleavage or the TCA cycle may affect susceptibility to ferroptosis. The major influencing factors are the AATs. SLC7A5 is required for the efficient growth of KRAS-mutant colorectal cancer (Najumudeen et al., 2021). Inhibition of glutamate oxaloacetate transaminase 1 sensitizes CRC cells to 5-fluorouracil (Hong et al., 2017).

NADPH is a substrate for NOX, which generates a superoxide anion. NOX inhibitors inhibit ferroptosis in human and plant cells (Dangol et al., 2019), and activation of NOX4 or NOX1 sensitizes cells to ferroptosis (Poursaitidis et al., 2017). The Hippo pathway effector TAZ also promotes ferroptosis by activating NOX4 and NOX2 in renal cell carcinoma and ovarian cancer cells, respectively (Yang et al., 2019; Yang W. H. et al., 2020). Loss-of-function NOX2 variants expressed in phagocytes and NOX1/DUOX2 variants expressed in intestinal epithelial cells are associated with very early onset IBD and pediatric and adult IBD (Stenke et al., 2019). Oxidative stress in the gut is considered a major contributor to the pathogenesis and progression of IBD. Lam et al. suggested a positive correlation between upregulated NOX and gastrointestinal inflammation (Lam et al., 2015). NOX acts through ROS to play multiple roles in regulating the intestinal barrier in homeostasis, infectious diseases, and intestinal inflammation (Aviello and Knaus, 2018). NADPH can be produced through various pathways, including the NADP-dependent isocitrate dehydrogenase (IDH), which can also generate NADPH (Pollak et al., 2007). SIRT2-dependent deacetylation of IDH1 suppresses CRC and liver metastases (Wang B. et al., 2020). IDH1/2 mutations are rare in CRC but commonly found in BRAF V600E-mutated CRC and colitis-associated CRC. However, further studies are needed to explore the clinicopathologic features and implications of targeted therapy for IDH1/2-mutated CRC (Huang et al., 2021).

Phospholipase A2 (PLA2) (Eaton et al., 2020) and lysophosphatidylserine lipase ABHD12 (Kathman et al., 2020) remove damaged fatty acyl chains from membrane lipids and negatively regulate ferroptosis in cancer cells. The PLA2 Group IIA (PLA2G2A) protein was detected in epithelial cells of the normal gastrointestinal tract, gallbladder, and pancreatic acinar cells. Intestinal glandular epithelial cells in Crohn’s disease and ulcerative colitis express abundant PLA2G2A, whereas inflammatory cells lack this protein. Tumor cells in colonic adenomas, carcinomas, and pancreatic ductal carcinoma express variable levels of PLA2G2A (Peuravuori et al., 2014). PAR-2-mediated activation of cPLA2 may be essential for intestinal myofibroblast proliferation, and upregulation of PAR-2 by inflammatory cytokines (such as TNF-α) may mediate this effect. PLA2G2A is increased in the colonic mucosa of patients with Crohn’s disease and ulcerative colitis (Minami et al., 1994). Paneth cells and columnar epithelial cells of the small and large intestinal mucosa synthesize PLA2-II at the sites of active inflammation in Crohn’s disease (Haapamäki et al., 1999). In addition, PLA2G2A acts as a modulator of gut microbiota (Taketomi et al., 2022). Furthermore, peroxiredoxin-6 (a bifunctional protein with PLA2 and peroxidase activity) inhibits ferroptosis mainly through its PLA2 activity (Lu et al., 2019). Overexpression of peroxiredoxin-6 is associated with aggressive biological behavior in advanced colon adenocarcinoma. Peroxiredoxin-6 is a key enzyme in the endogenous cellular response to oxidative stress/injury (Falidas et al., 2021). Molecular cloning studies revealed that approximately 50% of patients diagnosed with Crohn’s disease have antibodies against peroxiredoxin-6-like protein (Iizuka et al., 2012).

### 3.5 Ferroptosis influences intestinal diseases by affecting mechanotransduction

The cancer microenvironment is critical for tumorigenesis and cancer progression. The ECM can interact with tumors and stromal cells to promote cancer cell proliferation, migration, invasion, angiogenesis, and immune evasion. Cell membrane receptors and mechanical sensors can be activated by ECM and ECM-induced mechanical stimulation to modulate tumors. The piezoelectric, non-selective cation channels (Piezo), non-selective cation channels of the transient receptor potential (TRP) family, and Ca^2+^-activated chloride channels of the transmembrane protein 16/Anoctamin family (TMEM16/Ano) play an essential role in Ca^2+^-dependent cell death, including apoptosis and ferroptosis, under mechanical stimulation (Kunzelmann et al., 2019; Kim and Hyun, 2023; Otero-Sobrino et al., 2023).

Cells isolated from the ECM are considered the trigger for ferroptosis. The α6β4 integrins can protect cancer cells from erastin (an inducer of ferroptosis) and ECM detachment-induced ferroptosis by inhibiting the expression of ACSL4 (Brown et al., 2017). Consequently, iron overload produces abnormal ECM structures that lead to reduced cell proliferation, adhesion, and motility. Integrin α6β4 is highly expressed in several types of cancer (such as bladder, colon, ovarian, pancreas, prostate, and thyroid cancers) and is associated with poor prognosis (Tzanakakis et al., 2021). The association of integrin α6β4 with laminin substrates markedly promotes cancer cell adhesion, migration, invasion, proliferation, and tumorigenesis by activating the Rac1, PKC, and ERK signaling pathways (Tzanakakis et al., 2021). Overall, these findings suggest a certain link between intestinal tumors, ferroptosis, and α6β4 integrin.

PIEZO1 is highly expressed in patients with active Crohn’s disease and is positively correlated with Crohn’s disease activity index and fecal calprotectin levels. It is prominently expressed in the ileum (a part of the small intestine) and has been linked to intestinal inflammation (Liu et al., 2023). Once mechanical stimulation activates PIEZO1, the signals integrate with chemical pro-inflammatory signals to regulate macrophage function. PIEZO1 regulates important genes that produce pro-inflammatory mediators, such as IL-6, TNF-α, and prostaglandin E2 (Solis et al., 2019) by mediating Ca^2+^ influx. This influx leads to the activation of activator protein-1, production of endothelin-1, and stabilization of hypoxia-inducible factor 1α. In addition, PIEZO1 triggers an influx of calcium in intestinal epithelial cells, which leads to mitochondrial dysfunction and activates the NLRP3 inflammasome. The activation of the NLRP3 inflammasome further mediates intestinal inflammation (Liu et al., 2023). Xie et al. found that ferroptosis can activate NLRP3 inflammasomes and initial inflammatory responses *in vivo* through the HMGB1/TLR4 signaling (Xie et al., 2022). Inhibition of goblet cell-specific PIEZO1 in mouse models results in thinning of the intestinal mucus layer, increased production of inflammatory cytokines (such as CXCL1, CXCL2, and IL-6), and an increase in the number of pathogenic bacteria in the intestine. These factors can contribute to the exacerbation of inflammation (Liu et al., 2022).

Thermal TRP channels are associated with various digestive tumors. Overexpression of the TRPV1 has been implicated in the development of colon and pancreatic cancers (Huang et al., 2020; Wang et al., 2022). Capsiate, an intestinal metabolite, can activate TRPV1 to inhibit ferroptosis by upregulating the expression of GPX4, thereby reducing intestinal damage (Deng et al., 2021a). In addition, TRP channels can also influence intestinal diseases by regulating Rho-GTPase. TRPV2 can drive cAMP to increase the activation of PKA. cAMP signaling primarily activates human malignant tumors through PKA, which is associated with the cAMP–PKA–CREB signaling system. Ovarian, colorectal, and breast cancers can use the cAMP/PKA signaling pathway for invasion, migration, adhesion, clonal development, and other malignant characteristics (Thodeti et al., 2009). TRPV4 is an important regulator of endothelial-dependent vascular tone in multiple organs (e.g., bowel and lungs). TRPV4 promotes PKC-dependent RhoA activation, forming stress fibers and resulting in damage to organs, such as the intestines, lungs, and brain (Laragione et al., 2019). Thermal TRP can affect cell differentiation, proliferation, migration, adhesion, and death by regulating calcium ions, Rho-GTPase, and the cAMP/PKA signaling pathways, thereby affecting cell carcinogenicity and development.

TMEM16 proteins (also known as anoctamins) are involved in various functions, including ion transport, phospholipid scrambling, and regulation of other membrane proteins (Pedemonte and Galietta, 2014). The anoctamin 1 (ANO1/TMEM16A) protein (also known as DOG1) is a marker protein for gastrointestinal stromal tumors, and its role in cell proliferation and development of different types of malignant tumors has been extensively reported. Upregulation of ANO1 is associated with increased cell proliferation (Kunzelmann et al., 2019) and is essential for mucus secretion (Benedetto et al., 2019). Notably, the anticancer effect of the antihelminthic drug niclosamide may be related to ANO1 inhibition (Kunzelmann et al., 2019). Contrary to the proliferative effect of ANO1, ANO6 appears to help regulate different types of cell death. Melittin (present in bee venom) is a potent activator of anoctamin, which stimulates PLA2 (Sirianant et al., 2016; Schreiber et al., 2018). Activation of PLA6 or lipid peroxidation can be induced by melittin, leading to a significant increase in PLA or intracellular calcium (Sirianant et al., 2016). These changes activate ANO6, leading to ferroptosis in cancer cells. Notably, tumor cell lines killed by the ANO6 activator melittin were also driven to cell death through ferroptosis when exposed to erastin and RSL3.

## 4 Concluding remarks and future prospects

**FIGURE 4:**
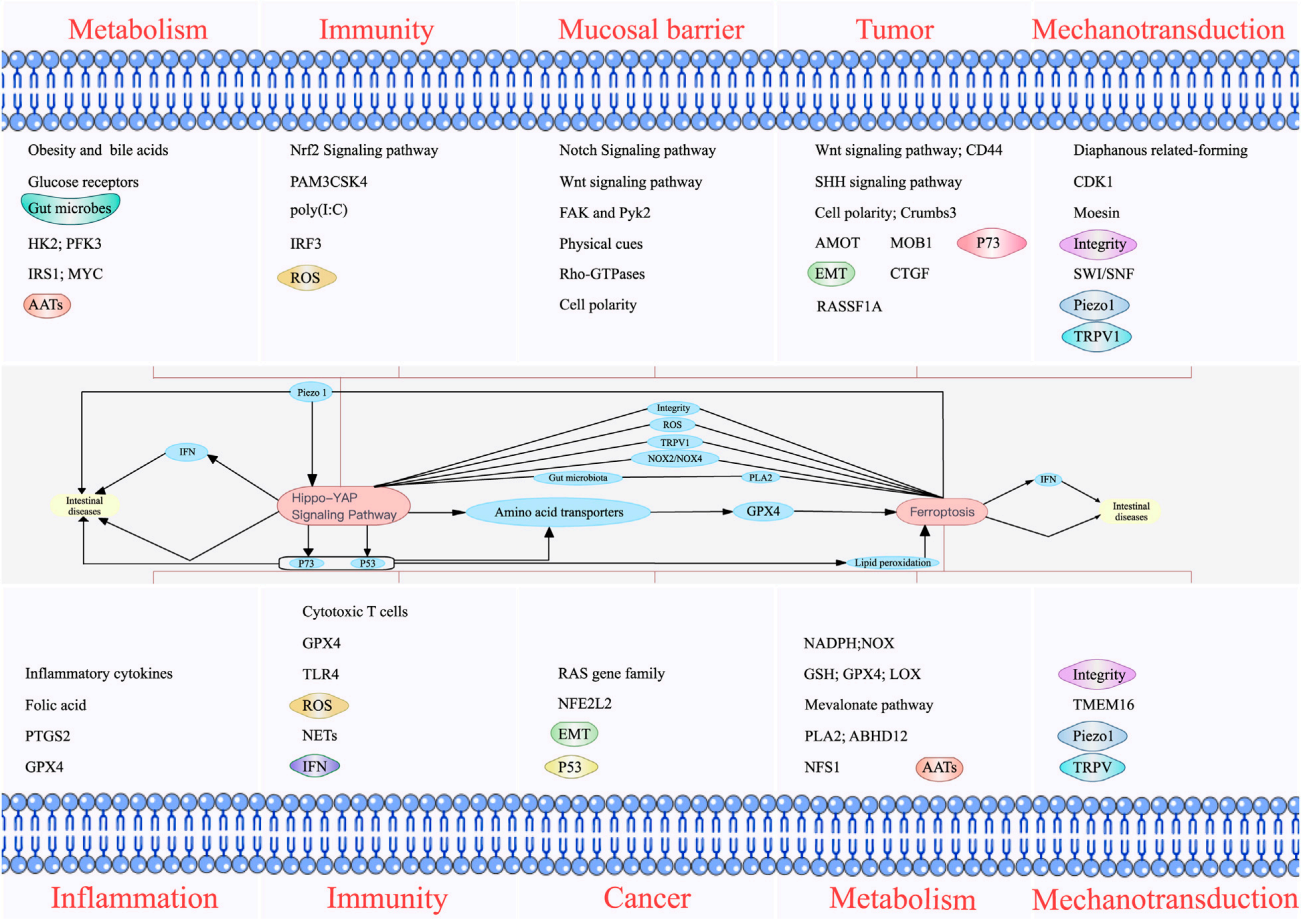
Individual and combined effects of the Hippo pathway and ferroptosis on intestinal diseases. The Hippo pathway influences intestinal diseases by modulating metabolism, immunity, mucosal barrier, and tumors. Ferroptosis also affects intestinal diseases by regulating inflammation, immunity, cancer, and metabolism. The Hippo pathway and ferroptosis interact with each other to influence intestinal diseases. Phospholipase A2 can directly influence ferroptosis and indirectly influence the Hippo pathway by affecting intestinal microbes. In addition, the Hippo pathway can affect ferroptosis by regulating NADPH oxidase 2/4, P53, and lipid peroxidation. The Hippo pathway can affect intestinal diseases by affecting P73. The Hippo pathway and ferroptosis can simultaneously affect intestinal diseases by affecting interferons.

Multiple factors upstream and downstream of the Hippo pathway and ferroptosis modulate intestinal diseases **(** Figure 4 **)**. Amino acid metabolism plays a significant role in mediating the effect of the Hippo pathway on ferroptosis. GPX4 is an essential regulator of ferroptosis, and the effects of ferroptosis on immunity, metabolism, inflammation, and cancer are associated with GPX4 and, the substrate of GPX4. GPX4 can affect the production of inflammatory factors, function of immune cells, metabolism of substances, and development of cancer. Therefore, it is an essential factor influencing intestinal diseases. The regulation of GPX synthesis discovered to date mainly focuses on regulating AATs. The AATs related to GPX4 include SLC7A11, SLC3A2, SLC1A5, and SLC7A5, and YAP can upregulate the expression of these AATs (Hansen et al., 2015; Gao et al., 2021; Kandasamy et al., 2021). Therefore, GPX4 is an important bridge between the Hippo pathway and ferroptosis. In addition, NOX2 and NOX4 can be regulated by TAZ, which promotes the occurrence of ferroptosis. Moreover, ROS, the ferroptosis inducers, can also activate MST and subsequently activate the Hippo pathway. The influence of ROS on intestinal diseases involves the participation of both ferroptosis and the Hippo pathway.

PLA2G2A, the negative regulator of ferroptosis, can act as a modulator of gut microbiota (Matallanas et al., 2007), and gut microbes can affect intestinal diseases through the Hippo pathway. Simultaneously, the Hippo pathway can affect the intestinal microbiota and intestinal diseases. Bile acid-activated receptors are highly expressed throughout the gastrointestinal tract and mediate bidirectional communication between the gut microbiota and the host immune system, which may also be linked to the Hippo pathway. Therefore, gut microbes may serve as a potential bridge between the Hippo pathway and ferroptosis.

P73 in the Hippo pathway can play an antitumor role, and P53 mediates the transcriptional repression of SLC7A11 to promote the ferroptosis of cancer cells (Vogel et al., 2014). P53 can also inhibit NOX-mediated lipid peroxidation in human CRC cells by directly binding to dipeptidyl peptidase 4 (Kunzmann et al., 2013). Lipid peroxidation is related to ferroptosis; therefore, the Hippo pathway can regulate intestinal diseases by inhibiting lipid peroxidation and subsequently inhibiting ferroptosis through P53. The genes and encoded proteins of P53 and P73 are similar, and knocking down P53 can inhibit YAP, suggesting that P53 can regulate both the Hippo pathway and ferroptosis.

EMT properties and cancer metastasis can be suppressed by downregulating YAP/TAZ (Najumudeen et al., 2021), and LYRIC, the positive regulator of EMT, can prevent cancer metastasis by inhibiting the expression of GPX4 and SLC3A2 to promote ferroptosis (Li et al., 2018). The downregulation of YAP/TAZ inhibits EMT, which may inhibit LYRIC and subsequently ferroptosis. However, the relationship between the Hippo pathway and ferroptosis in EMT remains to be further studied.

Squalene and cholesterol in the mevalonic acid pathway affect ferroptosis. OSCs can drive cancer cell proliferation and accelerate the onset of CRC (Kroschwald et al., 2018). Cholesterol accumulation reduces OSCs and accelerates CRC (Ou et al., 2016). In addition, OSCs promote CRC cell proliferation by activating the CYP24A1-mediated MAPK signaling (He et al., 2021). MAPK activates the Hippo pathway, which can affect intestinal diseases. Therefore, OSCs can regulate intestinal disease by regulating cholesterol accumulation, ferroptosis, and the Hippo pathway through MAPK.

The important immune molecule IFNs participate in the Hippo pathway and ferroptosis. IFNs and intestinal diseases affected by ferroptosis and the Hippo pathway are interlinked. However, the subtypes of IFN involved in the Hippo pathway and ferroptosis are different. The different effects of different subtypes of IFNs on the host may be related to the activated signaling pathways; however, the specific mechanism needs to be further studied.

The Hippo pathway participates in intestinal metabolism and thus influences intestinal diseases. The key enzyme PFK1 of glucose metabolism can regulate the activity of YAP. In addition, PFK1 can also regulate the ratio of AMP and ATP and activate AMPK (Hardie et al., 2012). In turn, AMPK can phosphorylate YAP (Mo et al., 2015). The lack of AMPK in intestinal epithelial cells has been found to inhibit intestinal epithelial cell repair *in vitro*; therefore, we can speculate that the role of AMPK in the repair of intestinal epithelial cells is related to YAP (Olivier et al., 2022).

In addition, we summarized the relationship between the Hippo pathway and inflammation and mucosa leading to intestinal diseases. For example, TLR ligands can interact with the Hippo pathway through MAPK, and TLR2/1 agonist PAM3CSK4 can induce differentiation of human and mouse monocytes into immunosuppressive M2 macrophages (Meng F. et al., 2016). The TLR3 agonist poly (I:C) prevents DSS-induced colitis (Zhong and Kyriakis, 2004). The effects of agonists on intestinal diseases may be caused by the regulation of the immune system through the Hippo pathway. YAP is critical to the repair of the intestinal mucosal barrier. When disrupted, F-actin inhibits phosphorylation of YAP (Deng et al., 2022). Therefore, YAP may be involved in intestinal diseases involving F-actin; however, further studies are needed.

Crumbs, CD44, and MOB can affect intestinal diseases by interacting with the Hippo pathway. The apical polarity protein Crumbs can promote cell delivery by promoting TAZ/YAP phosphorylation. In addition, the protein can regulate the expression of glycophospholipids on the plasma membrane to promote colon cancer cell migration. Therefore, we speculate that Crumbs and YAP interact in intestinal diseases. Many cancers are associated with abnormal Wnt or Hippo pathways, and the Wnt signaling pathway is upregulated in most CRCs. The CD44 variant expressed by ISCs activates the Wnt signaling to promote intestinal tumorigenesis (Song et al., 2019). In addition, CD44 may influence the development of intestinal tumors by activating the Hippo pathway. Therefore, we hypothesized that MOB can affect bowel cancer by activating YAP. MOB1 activates the LATS kinase in the Hippo pathway. A significant decrease in phosphorylation of MOB1 at Thr12 was found in cancer tissue samples, which was strongly associated with a decrease in the phosphorylation of YAP (Matallanas et al., 2007). Therefore, MOB and YAP may be closely associated with intestinal cancer.

We elaborated on the relationship between ferroptosis, folic acid, inflammation, metabolism, and intestinal diseases. Folic acid inhibits the ferroptosis. A meta-analysis suggested that folic acid supplementation had no significant effect on CRC risk (Kim et al., 2008). However, total folate intake reduces the risk of CRC and protects from this malignancy (Zhang et al., 2019). Therefore, the mitigating effect of folic acid on IBD and CRC may be related to the inhibitory effect of folate on ferroptosis; however, further research is needed.

Iron metabolism has also been implicated in ferroptosis (Ratajczak et al., 2021). The imbalance between M1 and M2 macrophages leads to inflammation, which is not only related to the function of the macrophages but also to the influence of macrophages on ferroptosis.

NOX is important in IBD as it regulates the intestinal barrier in infectious diseases and inflammation. NADPH is produced by NADP-dependent IDH (Elshazli et al., 2020). SIRT2-dependent deacetylation of IDH1 inhibits CRC (Rojo de la Vega et al., 2018). Therefore, IDH can modulate intestinal diseases by influencing NADPH synthesis and indirectly affecting ferroptosis.

The Hippo pathway and ferroptosis share common upstream regulatory factors in mechanotransduction and matrix stiffness, such as PIEZO1, integrins, and TRPV1. Therefore, mechanical signal transduction in the intestine can serve as an upstream regulator of the Hippo pathway and ferroptosis, thereby influencing intestinal diseases. Overall, it allows us to understand the link between the Hippo pathway and ferroptosis and their collective influence on intestinal diseases.

Most current studies on signaling pathways are at the multicellular level; however, even the expression levels of cellular genes in different parts of the same organ are different. For example, the Hippo activity from the villi to the base of the crypt shows a downward gradient. Future research is required to fill current knowledge gaps using advanced technologies. In addition, the gastrointestinal tract is an organ that directly communicates with the outside environment, and intestinal microbes play an essential role in maintaining the normal function of the intestinal tract. This feature is not present in the other organs. However, most of the studies reviewed in this paper have not considered the role of microbes. Moreover, the intestine is closely related to many other organs, such as the enterohepatic circulation and brain–gut axis. However, most of the current research only focuses on the intestine, and future research is required to reveal specific relationships of intestinal diseases with other organs.

## Figures and Tables

**Table:** 

**IBD**	Inflammatory Bowel Disease
**GPX4**	Glutathione peroxidase 4
**CRC**	colorectal cancer
**PFK1**	fructose 1, 6-bisphosphatase
**IRS1**	insulin receptor substrate 1
**AAT**	Amino acid transporter
**IFN**	Interferons
**IRF3**	IFN regulatory factor 3
**PAM3**	protospacer adjacent motif 3
**Nrf2**	Nuclear factor E2 related factor 2
**ISC**	intestinal stem cell
**FAK**	Focal adhesion kinase
**RASSF1A**	RAS-association domain family tumor suppressor protein 1A
**EMT**	Epithelial-to-mesenchymal transition
**AMOT**	Angiomotin
**GSH**	Glutathione
**PUFA**	polyunsaturated fatty acid
**ACSL4**	Acyl-CoA synthetase long-chain family member 4
**IRP-1 and IRP-2**	Iron regulatory proteins 1 and 2
**TFR1**	transferrin receptor 1
**NET**	neutrophil extracellular trap
**LOX**	Lipoxygenases
**OSCs**	Squalene epoxidases
**NOX**	NADPH oxidase
**IDH**	isocitrate dehydrogenase
**PLA2**	Phospholipase A2
**PLA2G2A**	phospholipase A2 Group IIA
**CDK1**	Cyclin-Dependent Kinase 1
**EHEC**	enterohemorrhagic *Escherichia coli*
**TRP**	transient receptor potential family
**TRPV1**	transient receptor potential cation channel V subfamily member 1
